# *Bacillus* CotA laccase improved the intestinal health, amino acid metabolism and hepatic metabolic capacity of Pekin ducks fed naturally contaminated AFB_1_ diet

**DOI:** 10.1186/s40104-024-01091-8

**Published:** 2024-10-10

**Authors:** Mingxin Ma, Qianqian Wang, Yanrong Liu, Guiming Li, Limeng Liu, Gaigai Wang, Yongpeng Guo, Shimeng Huang, Qiugang Ma, Cheng Ji, Lihong Zhao

**Affiliations:** 1 State Key Laboratory of Animal Nutrition and Feeding, Poultry Nutrition and Feed Technology Innovation Team, College of Animal Science and Technology, China Agricultural University, No. 2. Yuanmingyuan West Road, Beijing, 100193 People’s Republic of China; 2 Poultry Institute, Shandong Academy of Agricultural Sciences, Jinan, 250100 China; 3 College of Animal Science and Technology, Henan Agricultural University, Zhengzhou, 450046 China

**Keywords:** AFB_1_ residue, Aflatoxin, *Bacillus* CotA laccase, Duck, Intestinal barrier function, Liver metabolic enzyme

## Abstract

**Background:**

Aflatoxin B_1_ (AFB_1_) is a prevalent contaminant in agricultural products, presenting significant risks to animal health. CotA laccase from *Bacillus licheniformis* has shown significant efficacy in degrading mycotoxins in vitro test. The efficacy of *Bacillus* CotA laccase in animals, however, remains to be confirmed. A 2 × 2 factorial design was used to investigate the effects of *Bacillus* CotA laccase level (0 or 1 U/kg), AFB_1_ challenge (challenged or unchallenged) and their interactions on ducks. The purpose of this study was to evaluate the efficacy of *Bacillus* CotA laccase in alleviating AFB_1_ toxicosis of ducks.

**Results:**

*Bacillus* CotA laccase alleviated AFB_1_-induced declines in growth performance of ducks accompanied by improved average daily gain (ADG) and lower feed/gain ratio (F/G). *Bacillus* CotA laccase ameliorated AFB_1_-induced gut barrier dysfunctions and inflammation testified by increasing the jejunal villi height/crypt depth ratio (VH/CD) and the mRNA expression of tight junction protein 1 (*TJP1*) and zonula occluden-1 (*ZO-1*) as well as decreasing the expression of inflammation-related genes in the jejunum of ducks. Amino acid metabolome showed that *Bacillus* CotA laccase ameliorated AFB_1_-induced amino acid metabolism disorders evidenced by increasing the level of glutamic acid in serum and upregulating the expression of amino acid transport related genes in jejunum of ducks. *Bacillus* CotA laccase ameliorated AFB_1_-induced liver injury testified by suppressing oxidative stress, inhibiting apoptosis, and downregulating the expression of hepatic metabolic enzyme related genes of ducks. Moreover, *Bacillus* CotA laccase degraded AFB_1_ in digestive tract of ducks, resulting in the reduced absorption level of AFB_1_ across intestinal epithelium testified by the decreased level of AFB_1_-DNA adduct in the liver, and the reduced content of AFB_1_ residues in liver and feces of ducks.

**Conclusions:**

*Bacillus* CotA laccase effectively improved the growth performance, intestinal health, amino acid metabolism and hepatic aflatoxin metabolism of ducks fed AFB_1_ diets, highlighting its potential as an efficient and safe feed enzyme for AFB_1_ degradation in animal production.

**Graphical Abstract:**

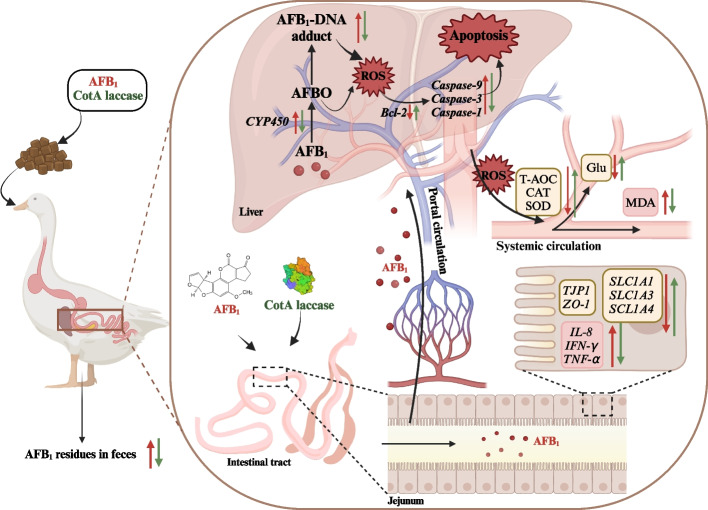

**Supplementary Information:**

The online version contains supplementary material available at 10.1186/s40104-024-01091-8.

## Background

Aflatoxins are noxious secondary metabolites that are produced by filamentous fungal species such as *Aspergillus flavus* and *Aspergillus parasiticus*, which mainly includes AFB_1_, aflatoxin B_2_ (AFB_2_), aflatoxin G_1_ (AFG_1_), aflatoxin G_2_ (AFG_2_), aflatoxin M_1_ (AFM_1_) and aflatoxin M_2_ (AFM_2_). Among aflatoxins, AFB_1_ is the most common, and also exhibits the highest toxicity, such as teratogenic, carcinogenic, and hepatotoxic toxicity [1–3]. Cereal crops are very susceptible to aflatoxins worldwide. The feed in China was universally found to be contaminated with AFB_1_, which was detected in 81.9%–100% of feedstuff and complete feed collected from different regions of China with the average levels ranging from 1.2–27.4 μg/kg during 2018–2020 [4]. Ducks that consume feed contaminated with AFB_1_ are at risk of poisoning, which can result in liver damage and immunotoxicity [5, 6]. The liver is the primary organ targeted by AFB_1_. Within the liver, phase I metabolism of AFB_1_ predominantly involves its conversion to AFB_1_-8,9-epoxide (AFBO), facilitated by the cytochrome P450 (CYP450) enzyme and then gives rise to metabolites such as aflatoxin Q_1_ (AFQ_1_) and AFM_1_ [7, 8]. Under phase II metabolism of AFB_1_, it can be catalyzed by glutathione-S-transferase (GST) to form aflatoxin 8,9-dihydro-8-(S-glutathionyl)-9-hydroxy aflatoxin B_1_ with lower toxicity [9]. Research has shown that AFB_1_ impaired growth performance, disrupted liver metabolism, triggered liver inflammation, and resulted in liver conditions such as swelling, steatosis, and bleeding in ducks [10, 11]. Therefore, there is a need for an effective strategy to mitigate the toxicity of AFB_1_ on ducks.

Previous studies summarized some approaches to detoxify AFB_1_ from food and feed, including physical, chemical, and biological approaches. Heat treatment, ultraviolet irradiation, and adsorption treatment are examples of physical procedures [12, 13], while ozone treatment is an example of chemical method. Due to the high cost, low efficiency, loss of nutrients, and chemical residue in food and feed caused by physical and chemical methods, both approaches have not been proven worthy of thorough detoxification and widely applied in animal production [14].

Detoxification of AFB_1_ by using microorganisms or enzymes can overcome the mentioned drawbacks and is considered an efficient, safe, and economical approach to detoxify AFB_1_ from the contaminated feed [14]. *Bacillus subtilis* ANSB060 isolated from fish gut can degrade AFB_1_, AFG_1_, and AFM_1_ in vitro, meanwhile this strain could resist unfavorable conditions within simulated gut environments [15]. The growth performance and meat quality of broilers were improved when the AFB_1_ naturally moldy diet was added with *Bacillus subtilis* ANSB060 [16]. Moreover, the combined probiotics with aflatoxin B_1_-degrading enzyme from *Aspergillus oryzae* could relieve the negative effect of AFB_1_ on chicken’s production performance and nutrient metabolic rates, suggesting a promising future for the application of AFB_1_-degrading enzymes [17, 18]. Presently, studies on AFB_1_-degrading enzymes primarily focus on validating AFB_1_ degradation in vitro, with limited in vivo experiments assessing the effectiveness and safety of AFB_1_-degrading enzymes in animal production [19–21].

CotA laccase from *Bacillus licheniformis* ANSB821 identified by our laboratory is highly thermostable and can degrade 70% AFB_1_ (2 µg/mL) within 30 min in vitro [22, 23], while the efficacy of *Bacillus* CotA laccase in animals remains to be confirmed. The current study aims to assess the AFB_1_ detoxification ability of *Bacillus* CotA laccase in ducks exposed to diets contaminated with AFB_1_.

## Materials and methods

### Experimental animals and diets

Experimental procedures were approved by the Laboratory Animal Welfare and Ethical Review Committee of China Agricultural University (approval No. AW41213202-1-3). A total of 192 male Pekin ducklings were purchased from Beijing Golden Star Duck Co., Ltd. (Beijing, China) and randomly assigned to 4 treatments with 6 replicate cages of 8 ducks each. A 2 × 2 factorial design was used to investigate the effects of *Bacillus* CotA laccase level (0 or 1 U/kg), AFB_1_ challenge (challenged or unchallenged) and their interactions on ducks. The 4 treatments were: (1) Control group (Control, basal diet); (2) CotA laccase group (CotA, basal diet with an additional 1 U/kg *Bacillus* CotA laccase); (3) AFB_1_ group (AFB_1_, moldy peanut meal taking the place of normal peanut meal); (4) AFB_1_ and *Bacillus* CotA laccase group (AFB_1_ + CotA, AFB_1_ diet with an additional 1 U/kg *Bacillus* CotA laccase). CotA laccase from *Bacillus licheniformis* ANSB821 was expressed in *Pichia pastoris GS115*, and freeze-dried in a vacuum for 24 h and then incorporated into the feed. The final AFB_1_ concentrations in the AFB_1_ group and the AFB_1_ + CotA group were set around 20 μg/kg, and the final AFB_1_ concentrations in the Control group and the CotA group were below 10 μg/kg. The determined concentrations of AFB_1_ in each of the four groups are presented in Table S1. Diets were pelleted in the KL-210 feed pellet extruder (Henan Qirun Machinery Equipment Co., Ltd., China). Ducks had ad libitum access to pellet feed and water, with continuous light. The experimental diets were formulated based on corn-soybean meal-peanut meal in accordance with the requirements of the National Research Council (NRC, 1994) [24]. Table 1 presents the composition and nutrients level of the basal diets.

**Table 1:** Composition and nutrient levels of the basal diets

Item, %	d 1–14	d 15–28
Corn	53.52	54.38
Soybean meal (46% CP)	14.94	9.82
Wheat flour	10.00	8.74
Peanut meal (48% CP)	15.00	15.00
Soybean oil	1.00	6.80
Dicalcium phosphate	2.32	2.27
Limestone	1.37	1.23
NaCl	0.30	0.30
Trace mineral premix^a^	0.15	0.15
Vitamin premix^b^	0.02	0.02
L-Lysine sulfate (70%)	0.73	0.63
L-Threonine (98.5%)	0.15	0.20
DL-Methionine (99%)	0.35	0.28
L-Tryptophan (98.5%)	0.00	0.03
Choline chloride (60%)	0.10	0.10
Phytase (10,000 IU/g)	0.03	0.03
Complex enzyme^c^	0.02	0.02
SUM	100.00	100.00
Nutrient and energy concentration^d^		
Metabolisable energy, kcal/kg	2,850	3,200
Crude protein	20.50	18.00
Crude fat	3.60	9.30
Crude ash	6.10	5.80
Crude fibre	3.00	2.70
Calcium	1.08	1.00
Effective phosphorus	0.48	0.46
Lysine	1.20	1.00
Methionine	0.60	0.50
Threonine	0.78	0.73
Tryptophan	0.20	0.20

^a^Mineral premix provided per kilogram of complete diet: copper, 8 mg; zinc, 75 mg; iron, 80 mg; manganese, 100 mg; selenium, 0.15 mg; iodine, 0.35 mg

^b^Vitamin premix provided per kilogram of complete diet: retinyl acetate, 24 mg; cholecalciferol, 6 mg; menadione, 2.65 mg; thiamine, 2 mg; riboflavin, 6 mg; cyanocobalamin, 0.025 mg; α-tocopheryl acetate, 20 mg; biotin, 0.0325 mg; folic acid, 1.25 mg; pantothenic acid, 12 mg; niacin, 50 mg

^c^Complex enzyme provided per kilogram of complete diet: xylanase, 25 U; cellulase, 1.5 U; β-mannanase, 3 U; β-glucanase, 4.5 U; lipase, 1 U; acid protease, 4 U; neutral protease, 4 U; α-amylase, 1 U; pectinase, 0.3 U

^d^Calculated value

### Sample collection

On d 28, one duck from each replicate close to the average body weight was selected for sample collection. Polypropylene tubes were used to collect blood samples from the wing veins. By dislocating the neck vertebrae and bleeding from the carotid artery, ducks were slaughtered. Subsequently, liver tissues and jejunal samples between the endpoint of the duodenal loop and Meckel’s diverticulum were collected, flushed, snap-frozen in liquid nitrogen, and fixed with a 10% neutral buffered formalin solution for histological analysis. All tissues were kept at −80 °C. Feces were collected from each replicate using sterile sampling bags and kept at −20 °C.

### Growth performance

On d 14 and 28, ducks were fed-deprived for 8 h to determine the body weight (BW). The average daily feed intake (ADFI), ADG, and F/G were calculated for d 1–14, 15–28 and 1–28, respectively. The data are presented as mean ± standard error of the mean (SEM) (*n *= 6).

### Histopathology of liver and jejunum

Fixed liver and jejunum tissues were embedded in paraffin, and tissue rings were sliced into 5-μm thickness, deparaffinized in xylene, rehydrated, and mounted on glass slides [25, 26]. Sections were stained by haematoxylin and eosin (H&E). The slides were photographed on a Pannoramic MIDI digital slide scanner (3DHISTECH Ltd., Budapest, Hungary). Stained tissue sections were examined using CaseViewer V 2.43 (3DHISTECH Ltd., Budapest, Hungary). 

### Transcriptional analysis

Total RNA was extracted from the liver and jejunum samples, then reverse transcription was performed using commercial kits (RC112, R223-01; Vazyme Biotech Co., Ltd., Nanjing, China) according to the manufacturer’s instructions. Two-step quantitative real-time PCR was performed with Taq Pro Universal SYBR qPCR Master Mix (Q712-02; Vazyme Biotech Co., Ltd., Nanjing, China) on a Real-Time PCR Detection Systems (CFX Connect™, Bio-Rad, Hercules, California, USA). The relative levels of mRNA expression were calculated using the 2^−ΔΔCT^ method, which normalized to the reference mRNA level of* GAPDH*. The values of the control group were used as a calibrator. The primers used in this study are listed in Table S2.

### Amino acid-targeted metabolome

Serum amino acids were analyzed by UHPLC-MS/MS. The UHPLC separation was performed by an Agilent 1290 Infinity II series UHPLC System (Agilent Technologies, Santa Clara, CA, USA). The assay development was performed on an Agilent 6460 triple quadrupole mass spectrometer) which was equipped with an AJS electrospray ionization (AJS-ESI) interface. The MRM data was analyzed using Agilent MassHunter Workstation Software (B.08.00).

### Serum biochemical analysis

The activities of aspartate aminotransferase (AST), alanine aminotransferase (ALT), catalase (CAT), superoxide dismutase (SOD), and the concentrations of total antioxidant capacity (T-AOC) and malondialdehyde (MDA) in serum were measured using commercial assay kits (C010-2-1, C009-2-1, A007-1-1, A001-3-2, A015-2-1, A003-1-2; Nanjing Jiancheng Bioengineering Institute, Nanjing, China) according to the manufacturer’s instructions.

### Determination of AFB_1_ residues and AFB_1_-DNA adduct levels

AFB_1_ residues in liver and feces were extracted using the total aflatoxin immunoaffinity column (Clover Technology Group, Beijing, China) according to manufacturer's instructions. The extracted samples containing AFB_1_ were measured by high-performance liquid chromatography (HPLC) [27]. In brief, sample containing AFB_1_ was filtered using RC 0.22 μm filter and 20 μL of volume was injected into the HPLC injection system. AFB_1_ detection was achieved using 360 and 440 nm as the wavelengths of excitation and emission, respectively. The mobile phase consisted of methanol–water (45:55, v/v), and the flow rate was 1 mL/min. The levels of AFB_1_-DNA adduct in liver were measured by the Elisa kit (HB253-NC, Hengyuan Biological Institute, Shanghai, China) according to the manufacturer’s instructions.

### Statistical analysis

The data was analyzed using GraphPad Prism V 8.0.1 (GraphPad Software, San Diego, California, USA). Two-way ANOVA was used to determine the main effects of *Bacillus* CotA laccase addition and AFB_1_ challenge, and their interaction. Tukey’s multiple comparison was used to separate means when interactive effects were significant (*P* < 0.05). Results are presented as the mean ± SEM.

## Results

### *Bacillus* CotA laccase alleviated AFB_1_-induced declines in growth performance of ducks

The growth performance of ducks is presented in Table 2. Results showed that there were significant interactions of *Bacillus* CotA laccase addition and AFB_1_ challenge on the BW at d 28, the ADG, and F/G of ducks during d 15–28, and d 1–28. AFB_1_ challenge significantly decreased the BW at d 28 and the ADG of ducks during d 15–28 and d 1–28, while increased the F/G (*P* < 0.05) of ducks during d 15–28 and d 1–28 compared with those in the Control group. The BW at d 28 and the ADG of ducks during d 15–28 and d 1–28 were significantly improved and the F/G were reduced (*P* < 0.05) in the AFB_1_ + CotA group compared with the AFB_1_ group.

**Table 2:** Effect of *Bacillus* CotA laccase addition and AFB_1_ challenge on the growth performance of ducks

**CotA laccase**	**0 U/kg**		**1 U/kg**			***P*-values**		
**AFB**_**1**_	**-**	** + **	**-**	** + **	**SEM**	**CotA laccase**	**AFB**_**1**_	**Interaction**
BW (d 14), g	502.33	488.37	501.83	525.83	22.47	0.2584	0.7554	0.2461
BW (d 28), g	1,720.88^a^	1,547.01^b^	1,714.88^a^	1,711.25^a^	31.05	0.0018	0.0006	0.0009^***^
d 1–14								
ADG, g	32.04	31.01	32.80	34.44	1.61	0.0807	0.7876	0.2557
ADFI, g	51.37	52.46	52.86	54.57	3.31	0.4503	0.5567	0.8956
F/G, g/g	1.60	1.69	1.61	1.59	0.06	0.2610	0.4701	0.2169
d 15–28								
ADG, g	87.04^a^	75.62^b^	86.65^a^	84.67^ab^	2.37	0.0178	0.0007	0.0107^*^
ADFI, g	151.67	162.89	155.02	157.70	6.23	0.8361	0.1301	0.3441
F/G, g/g	1.75^a^	2.15^b^	1.79^a^	1.86^a^	0.07	0.0267	0.0001	0.0038^**^
d 1–28								
ADG, g	59.54^a^	53.32^b^	59.72^a^	59.56^a^	1.12	0.0006	0.0006	0.0010^**^
ADFI, g	101.52	107.68	103.94	106.14	3.62	0.8651	0.1181	0.4483
F/G, g/g	1.68^a^	1.92^b^	1.70^a^	1.72^a^	0.05	0.0298	0.0015	0.0068^**^

*P*-values for the main effect of *Bacillus* CotA laccase addition, main effect of AFB_1_ challenge, and the interaction between the *Bacillus* CotA laccase addition and AFB_1_ challenge (^*^P < 0.05, ^**^P < 0.01, and ^***^P < 0.001). ^a,b^Different superscript letters within a row denote a significant difference (*P* < 0.05). All data are presented as mean ± SEM (*n* = 6)

*AFB*_1_ Aflatoxin B_1_,* BW* Body weight, *ADFI* Average daily feed intake, *ADG* Average daily gain, *F/G* Feed/gain ratio, *SEM* Standard error of the mean. “-” mean not added, “ + ” mean added

### *Bacillus* CotA laccase ameliorated AFB_1_-induced gut barrier dysfunctions and inflammation in ducks

**Fig. 1:**
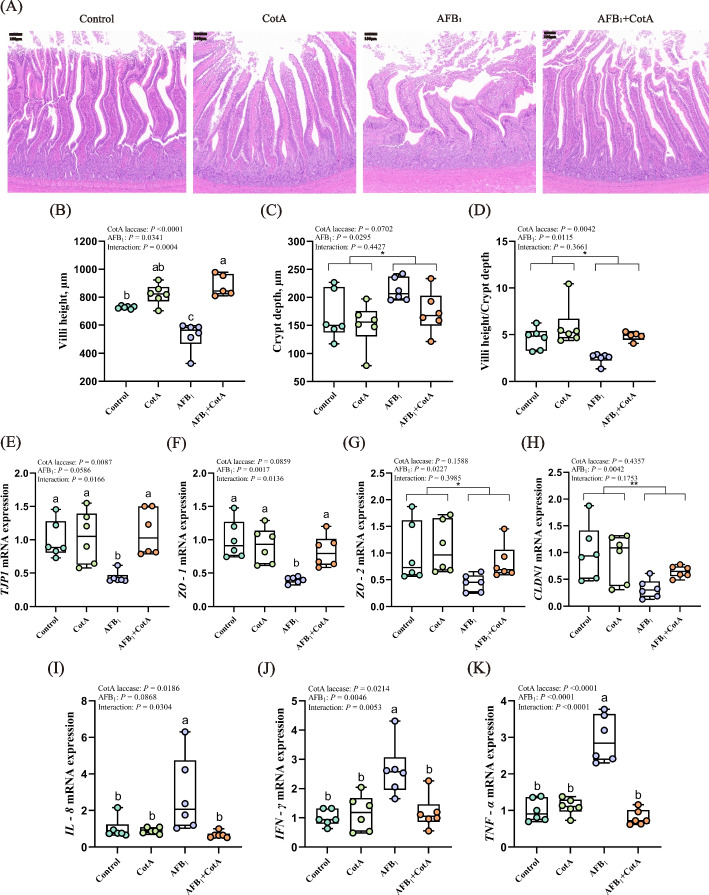
*Bacillus* CotA laccase ameliorated AFB_1_-induced gut barrier dysfunctions and inflammation in ducks. **A** H&E staining of jejunum in groups Control, CotA, AFB_1_, and AFB_1_ + CotA, scale bar = 100 μm; **B** Jejunal villi height; **C** Jejunal crypt depth; **D** Jejunal villi height/crypt depth; **E–H** The mRNA expression of *TJP1*, *ZO-1*, *ZO-2*, and *CLDN1* in the jejunum of ducks; **I–K** The mRNA expression of *IL-8*, *IFN-γ*, and *TNF-α* in the jejunum of ducks. All data are presented as mean ± SEM (*n* = 6). ^a−c^Different letters denote a significant difference (*P* < 0.05). ^*^P < 0.05, ^**^P < 0.01, *P*-value for the main effect of AFB_1_

H&E staining was utilized to observe the intestinal status of ducks in the four treatments. There was a significant interaction of *Bacillus* CotA laccase addition and AFB_1_ challenge on the jejunal villi height of ducks. In contrast with the Control group, the jejunum of ducks in the AFB_1_ group had severe pathological changes with the disappearance of villus architecture (Fig. 1A). The jejunal villi height in the AFB_1_ group was significantly reduced compared to that in the Control group, while the AFB_1_ + CotA group showed an observably higher villi height of jejunum compared with the AFB_1_ group. No interacting effect was observed between *Bacillus* CotA laccase levels and AFB_1_ challenge on jejunal crypt depth and VH/CD of ducks. AFB_1_ challenge markedly increased crypt depth and decreased the VH/CD of jejunum, while dietary addition of *Bacillus* CotA laccase presented a decreased tendency on crypt depth (*P* = 0.0702) and significantly improved the VH/CD of jejunum (Fig. 1B–D).

As to the mRNA expression of tight junction proteins, obvious interaction effects between *Bacillus* CotA laccase addition and AFB_1_ challenge were observed in the mRNA expression of *TJP1* and *ZO-1* in the jejunum of ducks. AFB_1_ challenge significantly decreased the mRNA expression of *TJP1* and *ZO-1* in the jejunum of ducks compared with the Control group, but these changes were markedly ameliorated in the AFB_1_ + CotA group (Fig. 1E and F).

The mRNA expression of zonula occluden-2 (*ZO-2*) and claudin 1 (*CLDN1*) was obviously decreased in the AFB_1_ treatment group compared to that in the group without AFB_1_ treatment (Fig. 1G and H).

As to the mRNA expression of inflammatory cytokines, there was obvious interaction effect between *Bacillus* CotA laccase addition and AFB_1_ challenge on the mRNA expression of interleukin 8 (*IL-8*), interferon gamma (*IFN-γ*), and tumor necrosis factor alpha (*TNF-α*) in the jejunum of ducks (Fig. 1I–K). The mRNA expression of *IL-8*, *IFN-γ* and *TNF-α* in the jejunum of ducks was observably increased in the AFB_1_ group compared to the Control group, but these changes were significantly alleviated in the AFB_1_ + CotA group.

In sum, these results indicated that *Bacillus* CotA laccase ameliorated AFB_1_-induced gut barrier dysfunctions and inflammation in ducks.

### *Bacillus* CotA laccase ameliorated AFB_1_-induced amino acid metabolism disorders in ducks

**Fig. 2:**
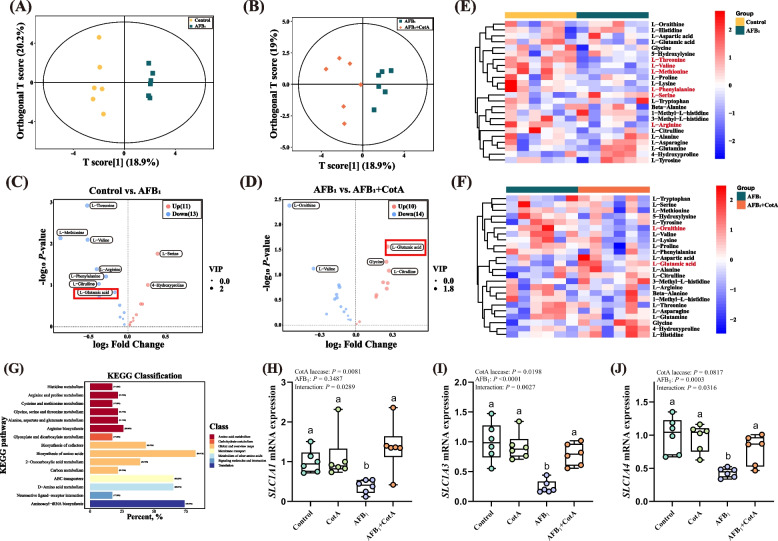
*Bacillus* CotA laccase ameliorated AFB_1_-induced amino acid metabolism disorders in ducks. **A** and **B** The OPLS-DA score plot and VIP values of the model of Control vs. AFB_1_ and AFB_1_ vs. AFB_1_ + CotA; **C** and** D** Volcano plots of amino acids in Control vs. AFB_1_ and AFB_1_ vs. AFB_1_ + CotA groups, blue represents low content while red represents high content; **E** and** F** Heat maps of amino acids concentrations in serum samples. Columns represent the samples (Control vs. AFB_1_ and AFB_1_ vs. AFB_1_ + CotA groups), and rows represent amino acids; **G** KEGG pathways enrichment analysis of AFB_1_ vs. AFB_1_ + CotA groups; **H–J** The mRNA expression of *SLC1A1*, *SLC1A3*, and *SLC1A4* in jejunum of ducks. All data are presented as mean ± SEM (*n* = 6). ^a,b^Different letters denote a significant difference (*P* < 0.05)

The amino acid metabolome analysis was performed to evaluate the impact of *Bacillus* CotA laccase on serum amino acid metabolism of ducks exposed to AFB_1_. Based on the OPLS-DA model (Fig. 2A), there was a clear separation in metabolites between the Control group and the AFB_1_ group, indicating that AFB_1_ treatment altered the serum metabolomics profile. And there was a clear separation of amino acid metabolites between the AFB_1_ group and the AFB_1_ + CotA group (Fig. 2B). A total of 24 amino acid metabolites were changed in the AFB_1_ group, including 11 upregulated metabolites and 13 downregulated metabolites compared to those in the Control group (Fig. 2C). The AFB_1_ + CotA group had 10 upregulated metabolites and 14 downregulated metabolites compared to the AFB_1_ group (Fig. 2D). The heatmap showed the distinct expression patterns of 24 metabolites in the serum of ducks between the Control group and the AFB_1_ group (Fig. 2E), as well as between the AFB_1_ group and the AFB_1_ + CotA group (Fig. 2F). Notably, compared with the Control group, glutamic acid level was lower in serum of ducks in the AFB_1_ group, while the AFB_1_ + CotA group reversed this change. KEGG classification analysis revealed that the biosynthesis of amino acids was the most enriched pathway among all the changed amino acid metabolite pathways in the AFB_1_ and AFB_1_ + CotA groups (Fig. 2G).

Additionally, we measured the mRNA expression of genes associated with glutamic acid transport in the jejunum of ducks. As shown in Fig. 2H–J, there was an obvious interaction effect between *Bacillus* CotA laccase addition and AFB_1_ challenge on the mRNA expression of solute carrier family 1 member 1 (*SLC1A1*), solute carrier family 1 member 3 (*SLC1A3*) and solute carrier family 1 member 4 (*SLC1A4*) in the jejunum of ducks. AFB_1_ exposure decreased the mRNA expression of *SLC1A1*, *SLC1A3,* and *SLC1A4* in the jejunum of ducks compared to the Control group, but these changes were significantly alleviated in the AFB_1_ + CotA group.

In sum, these results indicated that *Bacillus* CotA laccase ameliorated AFB_1_-induced amino acid metabolism disorders in ducks.

### *Bacillus* CotA laccase ameliorated AFB_1_-induced liver injury in ducks

**Fig. 3:**
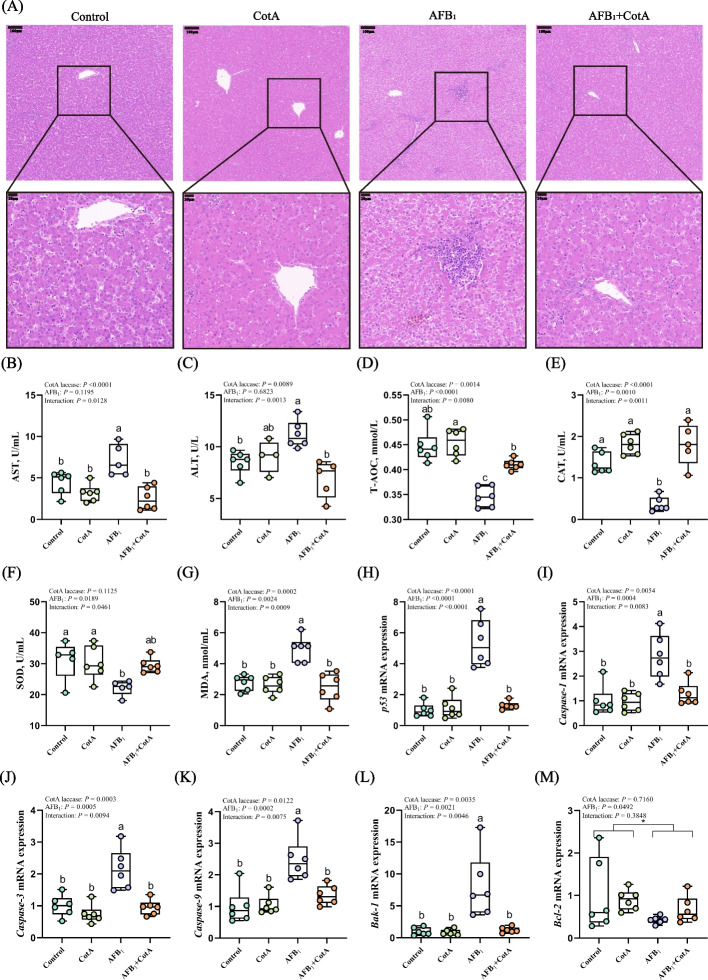
*Bacillus* CotA laccase ameliorated AFB_1_-induced liver injury in ducks. **A** H&E staining of liver sections in groups Control, CotA, AFB_1_, and AFB_1_ + CotA, scale bars are 100 μm and 20 μm, respectively; **B** Serum AST activity; **C** Serum ALT activity; **D** Serum T-AOC content; **E** Serum CAT activity; **F** Serum SOD activity; **G** Serum MDA content; **H–M** The mRNA expression of *p53*, *Caspase-1*, *Caspase-3*, *Caspase-9*, *Bak-1*, and *Bcl-2* in liver of ducks. All data are presented as mean ± SEM (*n* = 6). ^a−c^Different letters denote a significant difference (*P* < 0.05). ^*^P < 0.05, *P*-value for the main effect of AFB_1_

Histological analysis of liver was showed in Fig. 3A. In the AFB_1_ group, liver cell displayed unclear line arrangement and inflammatory cell infiltration, these damages were disappeared in the AFB_1_ + CotA group. To further investigate the status of liver injury, the serum activities of ALT and AST were measured. Results indicated that significant interactions were observed between *Bacillus* CotA laccase addition and AFB_1_ challenge on the activities of AST and ALT in serum of ducks. The activities of AST and ALT in the serum were significantly higher in the AFB_1_ group compared with those in the Control group, but these changes were significantly ameliorated in the AFB_1_ + CotA group (Fig. 3B and C).

The activities of antioxidant enzymes in the serum of ducks were determined to evaluate whether *Bacillus* CotA laccase could alleviate AFB_1_-induced oxidative damage (Fig. 3D–G). There were significant interactions between *Bacillus* CotA laccase addition and AFB_1_ challenge on the activities of CAT and SOD, and the concentrations of T-AOC and MDA in the serum of ducks. The lower activities of CAT and SOD, the lower concentration of T-AOC, and the higher concentration of MDA in the serum of ducks were observed in AFB_1_ group compared with the Control group (*P* < 0.05). *Bacillus* CotA laccase supplementation in the AFB_1_ diet reversed these changes compared with the AFB_1_ group (*P* < 0.05).

It’s widely accepted that oxidative damage could cause cell apoptosis in the body, so the mRNA expression of apoptosis related genes in liver was measured to evaluate whether *Bacillus* CotA laccase could alleviate the apoptosis caused by dietary AFB_1_. There were significant interactions between *Bacillus* CotA laccase addition and AFB_1_ challenge on the mRNA expression of tumor suppressor protein 53 (*p53*), cysteine-aspartic acid protease 1 (*Caspase-1*), cysteine-aspartic acid protease 3 (*Caspase-3*), cysteine-aspartic acid protease 9 (*Caspase-9*) and Bcl-2 antagonist/killer 1 (*Bak-1*) in the liver of ducks. The mRNA expression of *p53, Caspase-1, Caspase-3, Caspase-9,* and *Bak-1* in the liver of ducks in the AFB_1_ group was significantly increased compared to those in the Control group. Conversely, dietary *Bacillus* CotA laccase supplementation remarkably reversed those changes caused by AFB_1_ (Fig. 3 H–L). In addition, AFB_1_ exposure decreased the mRNA expression of B-cell lymphoma-2 (*Bcl-2*) in the liver of ducks (*P* < 0.05) (Fig. 3M).

All the results revealed that *Bacillus* CotA laccase supplementation in the AFB_1_ diet could ameliorate AFB_1_-induced liver injury, oxidative damage, and cell apoptosis in ducks.

### *Bacillus* CotA laccase neutralized hepatic metabolic enzyme changes induced by AFB_1_ in ducks

**Fig. 4:**
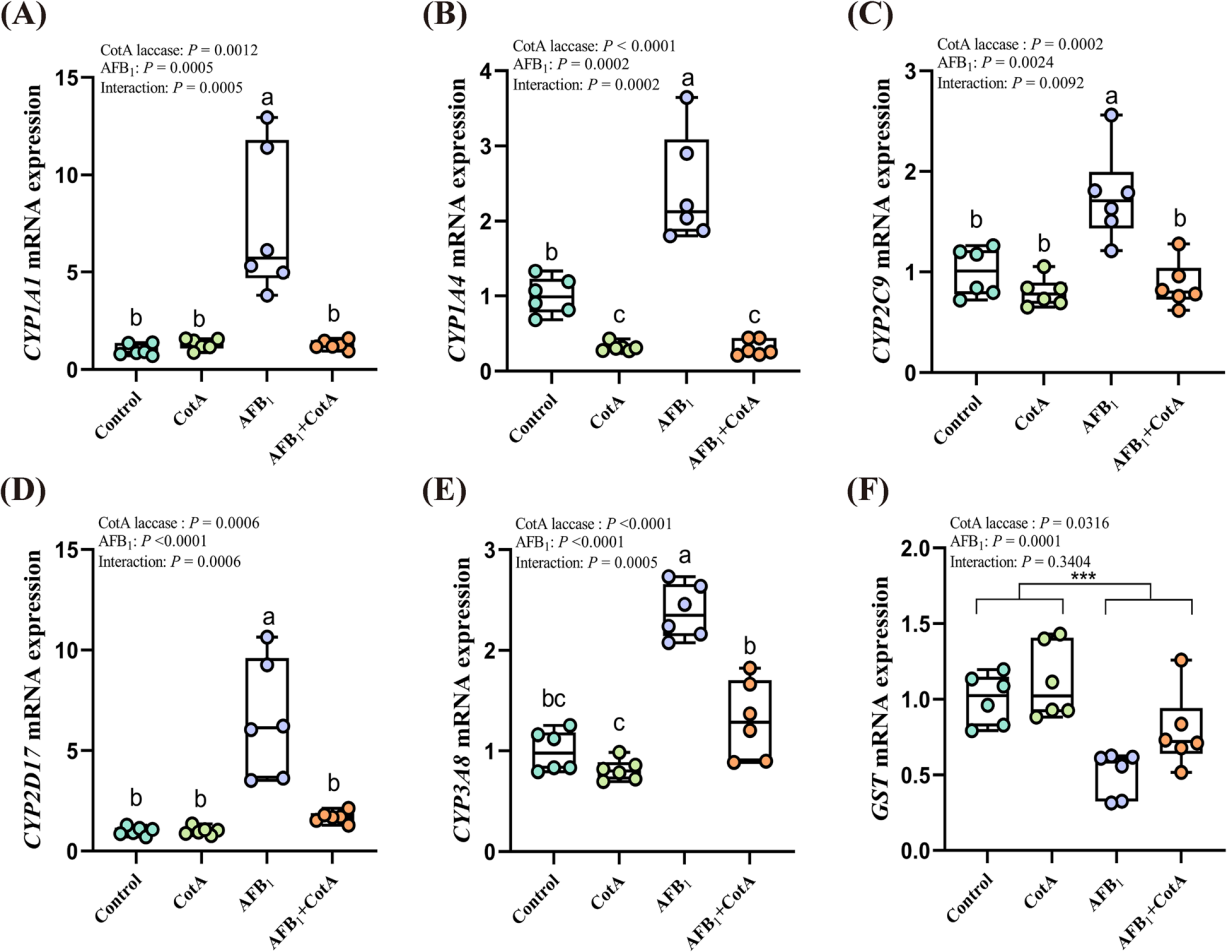
*Bacillus* CotA laccase neutralized hepatic metabolic enzyme changes in ducks induced by AFB_1_. **A–F** The mRNA expression of *CYP1A1*, *CYP1A4*, *CYP2D17*, *CYP2C9*, *CYP3A8,* and *GST* in liver of ducks. All data are presented as mean ± SEM (*n* = 6). ^a−c^Different letters denote a significant difference (*P* < 0.05). ^***^P < 0.001, *P-*value for the main effect of AFB_1_

The metabolic process of AFB_1_ in the liver was conducted by the phase I enzyme cytochrome P450 (CYP450), which could metabolize AFB_1_ to AFBO, then causing the toxicity to the body. There were significant interactions between *Bacillus* CotA laccase addition and AFB_1_ challenge on the mRNA expression of *CYP1A1*, *CYP1A4*, *CYP2D17*, *CYP2C9*, and *CYP3A8* in the liver of ducks (*P* < 0.05). AFB_1_ challenge enhanced the mRNA expression of *CYP1A1*, *CYP1A4*, *CYP2D17*, *CYP2C9*, and *CYP3A8* compared to the Control group (*P* < 0.05), while the mRNA expression of these genes was significantly downregulated in the AFB_1_ + CotA group compared with the AFB_1_ group (Fig. 4 A–E). In addition, AFB_1_ challenge decreased the mRNA expression of phase II enzyme *GST* in the liver of ducks (*P* < 0.05; Fig. 4 F), and *Bacillus* CotA laccase addition alleviated this change. These results suggested that *Bacillus* CotA laccase ameliorated AFB_1_-induced hepatic metabolic enzyme changes in ducks.

### *Bacillus* CotA laccase decreased AFB_1_-induced AFB_1_-DNA adduct formation in the liver and the contents of AFB_1_ residues in the liver and feces of ducks

**Fig. 5:**
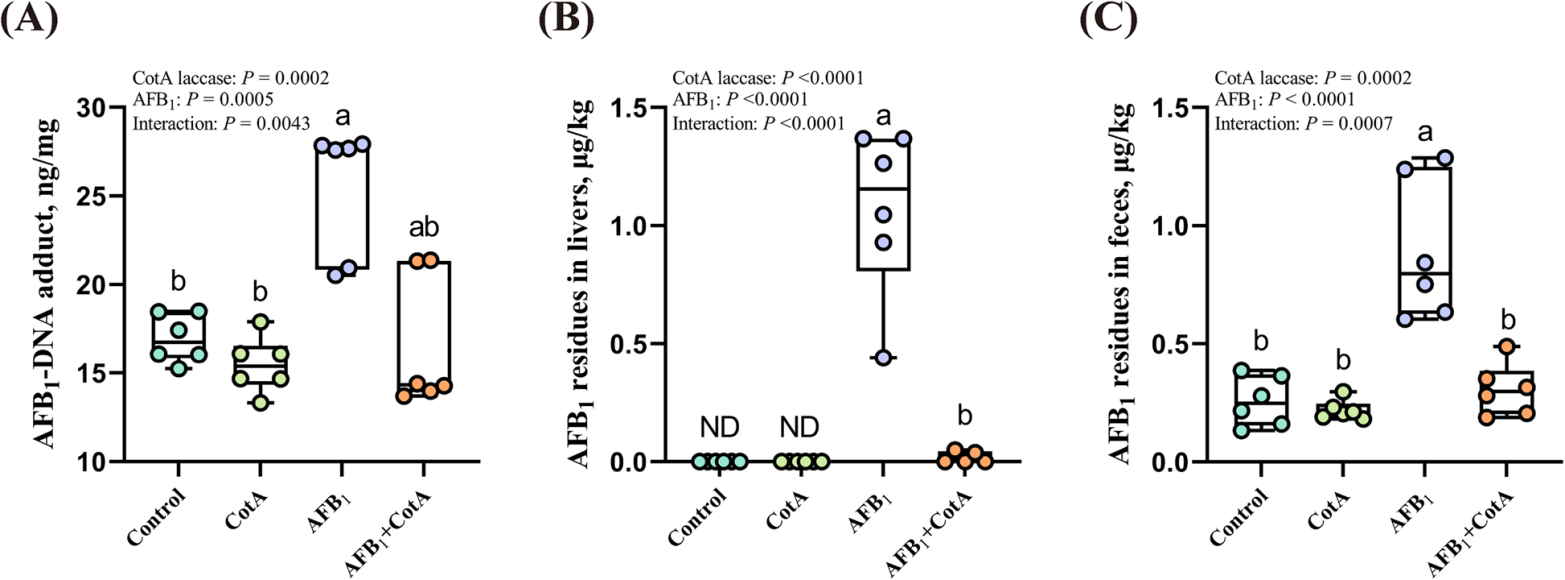
*Bacillus* CotA laccase reduced AFB_1_-induced AFB_1_-DNA adduct in liver and AFB_1_ residues in liver and feces of ducks. **A** AFB_1_-DNA adduct in liver; **B** AFB_1_ residues in liver (ND = not detected); **C** AFB_1_ residues in feces. All data are presented as mean ± SEM (*n* = 6). ^a,b^Different letters denote a significant difference (*P* < 0.05)

There were obvious interactions between *Bacillus* CotA laccase addition and AFB_1_ challenge on the content of AFB_1_-DNA adduct in the liver, and AFB_1_ residues in the liver and feces of ducks. AFB_1_ treatment significantly increased the content of AFB_1_-DNA adduct in the liver of ducks, and the residues of AFB_1_ in the liver and feces of ducks compared to those in the Control group. Whereas *Bacillus* CotA laccase supplementation in diet contaminated with AFB_1_ reduced the content of AFB_1_-DNA adduct in the liver of ducks, and the residues of AFB_1_ in the liver and feces of ducks compared with the diet contaminated with AFB_1_ without *Bacillus* CotA laccase (Fig. 5A–C).

## Discussion

Long term consumption of AFB_1_-contaminated feed by animals may result in the accumulation of AFB_1_ in animal products, thereby presenting a substantial health hazard to human consumers [28]. Hence, finding an effective AFB_1_ detoxification strategy and putting it into practical application is a crucial priority of the livestock industry. Enzymatic biotransformation is recognized as an efficacious and eco-friendly method for AFB_1_ detoxification, because enzymes can efficiently degrade AFB_1_ in the intestinal tract, then alleviate AFB_1_-induced damage in animals [17]. However, it is currently unconfirmed whether dietary *Bacillus* CotA laccase supplementation can alleviate the toxicity induced by AFB_1 _in ducks. In this study, AFB_1_-contaminated diets induced numerous adverse effects on ducks such as intestinal barrier damage, inflammatory responses, amino acid metabolism disruption, abnormal CYP450 enzyme metabolism in the liver, and compromised growth performance. Nonetheless, dietary supplementation of *Bacillus* CotA laccase could effectively mitigate these adverse effects caused by AFB_1_ in ducks.

Production performance serves as the primary indicator for assessing the health status of poultry. Research has demonstrated that dietary AFB_1_ exposure adversely impacts the growth performance of animals, as evidenced by reductions in ADFI, ADG, and feed conversion ratio [29–31]. This study unequivocally emphasized the toxic effects of dietary AFB_1_ at a concentration around 20 μg/kg on the growth performance of ducks, which was consistent with the previous research [32]. Nevertheless, this study proved that *Bacillus* CotA laccase effectively mitigated the toxicity induced by AFB_1_ and improved the growth performance of ducks, highlighting the practical application potential of *Bacillus* CotA laccase in the poultry industry.

The integrity of the intestinal barrier could protect the host from various pathogens, bacterial metabolites, and toxins [33]. The intestinal barrier includes physical, immunologic, and microbial components. The physical barrier is the first barrier to resist various damage to intestine [34]. Further, villus height, crypt depth, and VH/CD are crucial indicators of intestinal integrity [35]. Disruption of the intestinal barrier may trigger inflammatory responses, thereby posing a significant threat to animal health [36]. In this research, *Bacillus* CotA laccase demonstrated a capacity to mitigate the jejunal barrier damage induced by AFB_1_, as evidenced by improving the jejunal morphology, increasing the mRNA expression of tight junction proteins (*TJP1* and *ZO-1*), and decreasing the mRNA expression of inflammatory cytokines (*IL-8*, *IFN-γ*, and *TNF-α*). This suggested that *Bacillus* CotA laccase alleviated the intestinal barrier damage and inflammation induced by AFB_1_ in ducks.

Glutamic acid is crucial for the development of the intestinal mucosa, and plays an essential function in cellular metabolism, which benefits for the growth of young animals [37]. In this study, ducks exposed to AFB_1_ had lower level of glutamic acid in serum compared to ducks in the Control group. This finding aligns with the previous research in dairy goats indicating that AFB_1_ ingestion disrupts amino acid metabolism [38]. However, *Bacillus* CotA laccase ameliorated AFB_1_-induced amino acid metabolism disorders testified by increasing the level of glutamic acid in the serum of ducks. Non-essential amino acids, such as glutamine, glutamate, and aspartate, are primarily metabolized in the intestine. Amino acid transporter carriers facilitate the transport of these amino acids from the intestinal lumen, across the parietal membrane, and into the intestinal epithelium [39]. In this study, *Bacillus* CotA laccase supplementation alleviated AFB_1_-induced downregulation of mRNA expression of *SLC1A1*, *SLC1A3*, and *SLC1A4* in the jejunum of ducks. Besides, the amino acid transport didn’t exhibit a significant difference between the CotA group and the Control group. This finding suggests that *Bacillus* CotA laccase does not influence the absorption of micronutrients in the intestinal tract of animals. However, previous studies have indicated that certain adsorbents may bind essential minerals and nutrients present in the feed during the AFB_1_ detoxification process, potentially resulting in micronutrient deficiencies in animals [40].

Relevant studies have revealed that AFB_1_ exposure could lead to liver injury, including vacuolar degeneration and increased ALT and AST activities in the serum [41, 42], which is consistent with this study. What’s more, dietary *Bacillus* CotA laccase addition ameliorated AFB_1_ induced liver injury in ducks, which was proved by the decreased activities of ALT and AST in the liver and the serum. AFB_1_ also could damage the antioxidant capacity in animal, including the reduction of antioxidant enzyme activities and the increase of MDA level [43]. Antioxidant enzymes such as CAT and SOD are widely acknowledged as key defenders in cells, protecting body against oxidative damage. MDA is an important biomarker for assessing lipid peroxidation [44]. In this study, increased serum MDA concentration and decreased serum T-AOC concentration, CAT and SOD activities were observed in the AFB_1_ group, meanwhile the addition of *Bacillus* CotA laccase into the AFB_1_ diet alleviated the reduction of antioxidant capacity induced by AFB_1_.

Furthermore, the oxidative damage has the potential to cause cell apoptosis, which is associated with the activation of Caspase family [45, 46]. AFB_1_ treatment increased the mRNA expression of *p53, Caspase-1, Caspase-3, Caspase-9,* and *Bak-1*, which is consistent with previous evidence that AFB_1_ caused caspase-mediated apoptosis [47]. Notably, the addition of *Bacillus* CotA laccase into AFB_1_ diet significantly reduced the mRNA expression of these genes in ducks compared to the AFB_1_ diet. Thus, these findings suggested that *Bacillus* CotA laccase could mitigate AFB_1_-induced oxidant damage and cell apoptosis testified by enhancing antioxidant enzyme activity and reducing apoptosis-related gene expression.

The process of AFB_1_ metabolism mainly occurs in the liver, metabolizing AFB_1_ to AFBO by CYP450 enzymes [14]. Moreover, AFBO bonds with biomacromolecules like DNA, resulting in the formation of AFB_1_-DNA adduct [48]. AFB_1_-DNA adduct represents promising biomarkers for evaluating AFB_1_ exposure and AFBO production in animals [49]. In this study, the mRNA expression of *CYP1A1*, *CYP1A4*, *CYP2D17*, *CYP2C9*, and *CYP3A8* was downregulated in the AFB_1_ + CotA group compared to the AFB_1_ group, indicating that the addition of *Bacillus* CotA laccase into diet mitigated the hepatotoxic effects of AFB_1_. The decrease of AFB_1_-DNA adduct content in the liver of ducks in the AFB_1_ + CotA group further supported this finding. In the liver, AFB_1_ also undergoes a phase II metabolism mediated by GST, metabolizing AFBO to metabolites with lower toxicity [9]. The mRNA expression of *GST* in the liver of ducks in the group with AFB_1_ was significantly reduced. AFB_1_ can induce the excessive production of lipid peroxidation in the body, reduce the activity of antioxidant enzymes in the liver, and ultimately compromise the total antioxidant capacity of the body. However, the addition of *Bacillus* CotA laccase into the AFB_1_ diet significantly improved the mRNA expression of *GST* in the liver compared to the AFB_1_ diet. These findings collectively indicated that *Bacillus* CotA laccase had the strong detoxification capability in intestinal tract of animal, and reduced the concentration of AFB_1_ absorbed by enterocyte, which lead to the decreased levels of AFB_1_-DNA adduct in the liver and the residues of AFB_1_ in the liver and feces of ducks, thus maintaining the normal hepatic metabolism.

In summary, the current study firstly proved that *Bacillus* CotA laccase could alleviate AFB_1_-induced liver and intestinal toxicity in ducks. Further studies need to be carried out to investigate whether *Bacillus* CotA laccase can effectively alleviate the toxicity of livestock and poultry fed with diets contaminated with multiple mycotoxins, and reduce the residues of mycotoxins in animal products.

## Conclusion

*Bacillus* CotA laccase effectively improved the growth performance, intestinal health, amino acid metabolism and hepatic AFB_1_ metabolism, reduced the content of AFB_1_-DNA adduct in the liver and the residues of AFB_1_ in the liver and feces of ducks fed naturally contaminated AFB_1_ diet as it had the strong detoxification capability in intestinal tract of ducks, highlighting its potential as an efficient and safe feed enzyme for AFB_1_ detoxification in the livestock and poultry production.

## Supplementary Information

**Additional file 1:** **Table S1** The determined levels of AFB1 in diets; **Table S2** Sequences and product sizes of primers for target genes. 

## Data Availability

The datasets used during the current study are available from the corresponding author on reasonable request.

## Abbreviations

ADFI: Average daily feed intake
ADG: Average daily gain
AFB_1_: Aflatoxin B_1_
AFBO: Aflatoxin B_1_-8,9-epoxide
ALT: Alanine aminotransferase
AST: Aspartate aminotransferase
Bak-1: Bcl-2 antagonist/killer 1
Bcl-2: B-cell lymphoma-2
BW: Body weight
CAT: Catalase
Caspase-1: Cysteine-aspartic acid protease 1
Caspase-3: Cysteine-aspartic acid protease 3
Caspase-9: Cysteine-aspartic acid protease 9
CD: Crypt depth
CLDN1: Claudin 1
CP: Crude protein
CYP1A1: Cytochrome P450 family 1 subfamily A1
CYP1A4: Cytochrome P450 family 1 subfamily A4
CYP2C9: Cytochrome P450 family 2 subfamily C9
CYP2D17: Cytochrome P450 family 2 subfamily D17
CYP3A8: Cytochrome P450 family 3 subfamily A8
F/G: Feed/gain ratio
GAPDH: Glyceraldehyde 3-phosphate dehydrogenase
GST: Glutathione S-transferase
H&E: Haematoxylin and eosin
IFN-γ: Interferon gamma
IL-8: Interleukin 8
MDA: Malondialdehyde
p53: Tumor suppressor protein 53
SEM: Standard error of the mean
SLC1A1: Solute carrier family 1 member 1
SLC1A3: Solute carrier family 1 member 3
SLC1A4: Solute carrier family 1 member 4
SOD: Superoxide dismutase
T-AOC: Total antioxidant capacity
TJP1: Tight junction protein 1
TNF-α: Tumor necrosis factor alpha
VH: Villi height
ZO-1: Zonula occluden-1
ZO-2: Zonula occluden-2
