# Supplementary material for: Bacillus CotA laccase improved the intestinal health, amino acid metabolism and hepatic metabolic capacity of Pekin ducks fed naturally contaminated AFB1 diet
Source: J Anim Sci Biotechnol. 2024 Oct 10;15:138. doi: 10.1186/s40104-024-01091-8 (PMC11465776; doi:10.1186/s40104-024-01091-8)
Supplement: Supplementary file 1 — Additional file 1: Table S1 The determined levels of AFB1 in diets; Table S2 Sequences and product sizes of primers for target genes. [file 40104_2024_1091_MOESM1_ESM.docx]

**Additional file 1**

**Table S1** The determined levels of AFB_1_ in diets

| **Item** | **Treatment** | | | |
| --- | --- | --- | --- | --- |
|  | **Control** | **CotA** | **AFB_1_** | **AFB_1_+CotA** |
| AFB_1_, μg/kg | 8.716 | 7.743 | 19.131 | 16.688 |

Abbreviations: CotA, CotA laccase; AFB_1_, aflatoxin B_1_

**Table S2** Sequences and product sizes of primers for target genes

| **Transcripts** | **Accession number** |  | **Gene sequence (5´→3´)** | **Product length, bp** |
| --- | --- | --- | --- | --- |
| *p53* | XM_038174815.1 | F | ACTGCTACGTCGCGGCTCTC | 199 |
|  |  | R | CGCTGGCAAGGCTGGTGAAC |  |
| *Caspase-1* | XM_027446016.1 | F | GCGGAACCAAGAGCAGAGATGAG | 130 |
|  |  | R | CCACGGCAGGACTGGATAATAACC |  |
| *Caspase-3* | XM_021279218.3 | F | TGAGGCAGACAGTGGACCAGATG | 156 |
|  |  | R | CTGCATTCCGCCAGGAGTAATAGC |  |
| *Caspase-9* | XM_038166520.1 | F | TGGATTGCGATTCACCCGAAGATG | 83 |
|  |  | R | ATTACCCGAGGGAGCCTGGAAAG |  |
| *Bcl-2* | XM_005028719.1 | F | ACCTGGTTCTGAATAAGTGGGAT | 187 |
|  |  | R | GGTTGTCTTCTCAGTGTTGCCT |  |
| *Bak-1* | XM_005026830.2 | F | CAGGAGAGAGAAGAGAGCG | 206 |
|  |  | R | TTGATGAAGTAATCGTAGGC |  |
| *GST* | XM_005009201.5 | F | TGCTGTGCTTTCTGGGTTTC | 190 |
|  |  | R | AGGCCCGAAATACCAGGAAA |  |
| *CYP1A1* | NM_205147.1 | F | AGGACGGAGGCTGACAAGGTG | 104 |
|  |  | R | AGGATGGTGGTGAGGAAGAGGAAG |  |
| *CYP1A4* | XM_027466425.2 | F | AGCACATCAGGGACATCACA | 172 |
|  |  | R | TACATGAGGCACCAGGACAG |  |
| *CYP2C9* | XM_027460066.2 | F | GGTTTGTGTTGTTTGCCTGC | 168 |
|  |  | R | ACTGGCCCATACTCTTTGCT |  |
| *CYP2D17* | XM_038181717.1 | F | TCTTCACCACTCTCCTGCAG | 196 |
|  |  | R | GCCTAGACCTGCTGCATTTC |  |
| *CYP3A8* | XM_005020119.4 | F | GGCCAAACCCAGATGAGTTC | 208 |
|  |  | R | GCTTGAGAGGGACCTGAGTT |  |
| *TJP1* | XM_013104936.1 | F | TCAGCGAGATGAACGAGCC | 189 |
|  |  | R | TCTGAAGGCTCTGACCTCTGG |  |
| *CLDN1* | XM_013108556.1 | F | GGCATCATATTCAGCACCTTC | 134 |
|  |  | R | GCCTTACGCACTACATCTTGG |  |
| *ZO-1* | XM_013093747.1 | F | ACGCTGGTGAAATCAAGGAAGAA | 255 |
|  |  | R | AGGGACATTCAACAGCGTGGC |  |
| *ZO-2* | XM_005019888.2 | F | ACAGTGAAAGAAGCTGGCGTAG | 131 |
|  |  | R | GCTGTATTCCCTGCTACGGTC |  |
| *TNF-α* | XM_005027491.5 | F | ACAGCCTATGCCAACAAG | 107 |
|  |  | R | TACAGGAAGGGCAACTCA |  |
| *IL-8* | NM_001310420.1 | F | TGAGAGAAACACCCGAGGAGAGC | 93 |
|  |  | R | ACAGCGAAGATAAGAACACCGATGC |  |
| *IFN-γ* | XM_013106209.1 | F | TCGTGGAACTGTCAAACCTTCA | 140 |
|  |  | R | TTGGAAGTCGAAGTCTCCACC |  |
| *SLC1A1* | NM_001310783.1 | F | GCCTTGCAATCCATTCCACT | 140 |
|  |  | R | AGGCAAAGTTGCTGAACTGG |  |
| *SLC1A3* | XM_027471993.2 | F | ACTTCGGGCAGATCATCACA | 227 |
|  |  | R | GAGATGCTCCACAATGCCAG |  |
| *SLC1A4* | KU048810.1 | F | GGAGCTAAAAGAAGTCGGCG | 221 |
|  |  | R | ATCCAGCCCTTGTTAGCACT |  |
| *GAPDH* | XM_038180584.1 | F | CTACTCATGGCCACTTCCGG | 536 |
|  |  | R | GGTAAGCTTCCCGTTGAGCT |  |

Abbreviations: *F*, forward; *R*, reverse. *p53*, tumor suppressor protein53; *Caspase-1*, Cysteine-aspartic acid protease 1; *Caspase-3*, Cysteine-aspartic acid protease 3; *Caspase-9*, Cysteine-aspartic acid protease 9; *Bcl-2*, B-cell lymphoma-2; *Bak-1*, BCL2 antagonist/killer 1; *GST*, glutathione S-transferase; *CYP1A1*, cytochrome P450 family 1 subfamily A1; *CYP1A4*, cytochrome P450 family 1 subfamily A4; *CYP2C9*, cytochrome P450 family 2 subfamily C9; *CYP2D17*, cytochrome P450 family 2 subfamily D17; *CYP3A8*, cytochrome P450 family 3 subfamily A8; *TJP1*, tight junction protein 1; *CLDN1*, claudin 1; *ZO-1*, zonula occluden-1; *ZO-2*, zonula occluden-2; *TNF-α*, tumor necrosis factor alpha; *IL-8*, interleukin 8; *IFN-γ*, interferon gamma; *SLC1A1*, solute carrier family 1 member 1; *SLC1A3*, solute carrier family 1 member 3; *SLC1A4*, solute carrier family 1 member 4
